# Stroÿma-Directed Molecular Targeted Therapy in Gastric Cancer

**DOI:** 10.3390/cancers3044245

**Published:** 2011-12-08

**Authors:** Yasuhiko Kitadai, Michiyo Kodama, Kei Shinagawa

**Affiliations:** Department of Gastroenterology and Metabolism, Hiroshima University Graduate School of Biomedical Sciences, 1-2-3 Kasumi Minami-ku, Hiroshima 734-8551, Japan; E-Mails: edorami@d4.dion.ne.jp (M.K.); shinagawakei@hiroshima-u.ac.jp (K.S.)

**Keywords:** gastric cancer, stroma, platelet-derived growth factor receptor (PDGFR), carcinoma-associated fibroblast (CAF)

## Abstract

Recent studies in molecular and cellular biology have shown that tumor growth and metastasis are not determined by cancer cells alone, but also by a variety of stromal cells. Tumor stroma contains abundant extracellular matrix and several types of cells, including carcinoma-associated fibroblasts (CAFs), endothelial cells, pericytes and inflammatory cells including macrophages. In gastric cancer tissues, tumor cells express platelet-derived growth factor (PDGF)-B. Stromal cells, including CAFs, pericytes and lymphatic endothelial cells, express PDGF receptor (PDGFR)-β. Administration of PDGFR tyrosine kinase inhibitor significantly decreases stromal reaction, lymphatic vessel area and pericyte coverage of tumor microvessels. Administration of PDGFR tyrosine kinase inhibitor in combination with cytotoxic chemotherapeutic drug(s) impairs the progressive growth and metastasis of gastric cancer. Activated stroma might serve as a novel therapeutic target in cases of gastric cancer.

## 1. Introduction

Gastric cancer is the world's fourth most common malignancy and the second leading cause of cancer death. The highest incidences are seen in Eastern Asia, and the lowest are observed in North America [1]. Regional variations in part reflect differences in dietary patterns and the prevalence of *Helicobacter pylori* infection [1], which is an important etiological factor for the occurrence of non-cardia gastric adenocarcinoma [1]. Epidemiologic studies have indicated that infection with *H. pylori* is a risk factor for gastric cancer, and, in 1994, the WHO/IARC classified this bacterium as a definite biologic carcinogen [2]. In addition, *H. pylori* inoculation into the stomach of Mongolian gerbils was shown to be associated with the occurrence of chronic gastritis, intestinal metaplasia and adenocarcinoma [3,4]. Chronic mucosal inflammation induced by *H. pylori* infection is thought to contribute significantly to the pathogenesis of atrophic gastritis, intestinal metaplasia, dysplasia, and gastric cancers.

Conventional therapies for gastric cancer include endoscopic treatment, surgery and chemotherapy, but the prognosis for advanced-stage disease with metastasis remains poor. New ideas for therapeutic strategies are needed, but development of novel strategies depends on detailed understanding of cancer biology, especially at the molecular level. A large number of genetic and epigenetic alterations in oncogenes, tumor suppressor genes, cell cycle regulators and DNA repair genes as well as genetic instability drive the multi-step process of gastric carcinogenesis [5]. In addition, the molecular events that characterize gastric cancers differ, depending on the histologic type, whether intestinal- or diffuse-type [5,6].

Recent studies have shown that tumor growth and metastasis are determined not only by cancer cells themselves, but also by a variety of stromal cells. The stroma constitutes a large part of most solid tumors, and tumor-stromal cell interaction contributes functionally to tumor growth and metastasis [7,8]. Tumor stroma contains many types of cells, including activated fibroblasts, vascular and lymphatic endothelial cells, pericytes (mural cells) and inflammatory cells such as macrophages. It has become clear that activated fibroblasts in cancer stroma are prominent modifiers of tumor progression, and they express several mesenchymal markers such as α-smooth muscle actin, fibroblast activation protein and vimentin; they are therefore called carcinoma-associated fibroblasts (CAFs) or myofibroblasts [9]. However, the mechanisms that regulate activation of fibroblasts and their accumulation in tumors and the precise origin of these CAFs are not fully understood. Herein, we discuss the importance of tumor-stromal cell interaction in the growth and metastasis of human gastric cancer and the possibility of stroma-oriented therapy to reduce the risk of cancer metastasis, focusing mainly on CAFs.

## 2. CAFs in the Tumor Microenvironment

Tumor tissues contain a heterogeneous population of fibroblasts and other cells of mesenchymal origin that originate from both the surrounding tissue and bone marrow [10]. Fibroblasts are the most abundant cell type in connective tissues and form the structural framework of tissues by synthesizing extracellular matrix (ECM). Under normal conditions, fibroblasts are in an inactive quiescent state. However, they become activated in wound healing and fibrosis, both of which require tissue remodeling. Stroma rich in myofibroblasts is termed “reactive stroma”, and it characterizes many invasive carcinomas including those of the breast, pancreas, colon and stomach because of their similarity to granulation tissue [11]. Once the wound healing process is completed, most of the myofibroblasts are eliminated from the granulation tissue by means of apoptosis; however, myofibroblasts in tumor stroma, *i.e.*, CAFs, are not eliminated by apoptosis. Therefore, tumors have been referred to as “wounds that never heal” [12]. CAFs have gene expression profiles that are distinct from those of normal fibroblasts [13], and the cells acquire a modified phenotype, similar to that of fibroblasts associated with wound healing. Normal fibroblasts are reported to inhibit progression of cancer [14,15]. However, numerous studies have provided evidence that CAFs promote tumor growth [16,17].

CAFs synthesize a variety of fibrillar ECM components, such as type-I, type-III and type-V collagen and fibronectin [18,19]. CAFs are known to modulate tumorigenic properties of neoplastic cells, including their proliferative, apoptotic and angiogenic properties [20], and they are thought to play a central role in the complex process of tumor-stroma interaction and consequent tumorigenesis. In experiments involving coinjection of CAFs and tumor cells CAFs promoted tumor growth [21]. Moreover, recent studies revealed extensive changes in the phenotype, and even the genotype, of CAFs compared with their normal counterparts [22]. Lieubeau *et al.* reported that progressive tumor growth correlates with proliferation of myofibroblasts, whereas regression of tumors is linked to the presence of a fibrous capsule, suggesting that the presence of myofibroblasts contributes to the growth of tumor cells [23]. Other studies have shown that a poor prognosis in cases of colorectal carcinoma is associated with abundance of CAFs or increased expression of fibroblast activation protein [24,25]. Experiments have shown that CAFs can affect sensitivity of pancreatic carcinoma cells to chemo- or radio-therapy; the tumor cells become less sensitive to chemotherapy when co-cultured with CAFs or grown in fibroblast-conditioned medium [26].

## 3. Bone Marrow-Derived MSCs as an Origin of CAFs

Although CAFs have been implicated in important aspects of solid tumor biology including tumor growth, angiogenesis and metastasis, the precise origins of CAFs are not clear [27]. CAFs are highly heterogeneous and are thought to be a mixed population derived from different sources. The main progenitors of CAFs seem to be resident fibroblasts [17]; CAFs can also originate from mural cells including pericytes and vascular smooth muscle cells [11], endothelial cells [28] and bone marrow-derived cells including various stem cells [29]. Epithelial-to-mesenchymal transition (EMT) of cancer cells and endothelial-to-mesenchymal transition (EndMT) may also account for CAFs that are present in tumors.

The two main types of stem cells in the bone marrow are hematopoietic stem cells and mesenchymal stem cells (MSCs). MSCs can be defined according to their ability to self-renew and differentiate into tissues of mesodermal origin, including bone, cartilage, muscle and adipose and connective tissues [30]. MSCs are reported to migrate to sites of tissue injury and sites of inflammation as well as to stroma in solid tumors, where they interact with tumor cells [31]. The interaction between MSCs and tumor cells may occur within the bone marrow microenvironment, a niche in which cancer cells can survive [32]. However, it has been shown that cancer cells at the primary site release specific factors that induce MSC mobilization and recruitment to stroma in solid tumors [31], where they interact with tumor cells within the tumor microenvironment and differentiate into CAFs [33]. Several studies have implicated molecules such as stromal-cell-derived factor (SDF)-1/CXCR4, monocyte chemoattractant protein (MCP)-1/CCR2 and platelet-derived growth factor (PDGF) in the tumor-homing ability of MSCs [34-36]. In an *in vitro* experiment, MSCs exposed to tumor-conditioned medium over a prolonged period of time assumed a CAF-like myofibroblastic phenotype, promoting tumor cell growth both *in vitro* and *in vivo* [37].

Guo *et al.* [38] recently constructed a mouse model of gastric cancer (Gan mouse model) by simultaneous activation of prostaglandin E2 and Wnt signaling in the gastric mucosa. Microvessel density increased significantly, and the expression of VEGF-A was induced predominantly in the stromal cells of gastric tumors in the model. Moreover, the investigators showed by bone marrow transplantation experiments that a subset of gastric myofibroblasts is derived from bone marrow [38]. We examined whether circulating MSCs have the ability to migrate to the stroma of orthotopic gastric tumors. After injection of PKH-labeled MSCs into the tail veins of TMK-1 tumor-bearing mice, MSCs were detected specifically in the tumor stroma at the primary site. In contrast, MSCs were not detected in non-cancerous tissues. In addition, we found that commingled MSCs were functionally incorporated into the stroma of orthotopic tumors, where they expressed CAF markers α-smooth muscle actin, PDGF receptor (PDGFR)-β and fibroblast activation protein [33]. Thus, the interaction between MSCs and cancer cells may lead MSCs to differentiate into CAFs. Tumor cells mixed with MSCs and implanted orthotopically resulted in a greater tumor volume and lower survival than did implantation of tumor cells alone. From a clinical study, Worthley *et al.* [39] reported recently that bone marrow-derived cells differentiated into CAFs in human gastric cancers that developed in female recipients of male allogeneic (sex-mismatched transplantation) stem cells. However, the precise bone marrow cell type that gives rise to CAFs remains unclear. Very recently, it was shown in a mouse model of inflammation-induced gastric cancer that at least 20% of CAFs originated from bone marrow-derived MSCs [40].

## 4. Growth Factors for Stromal Components in Gastric Cancer

**Figure 1.**
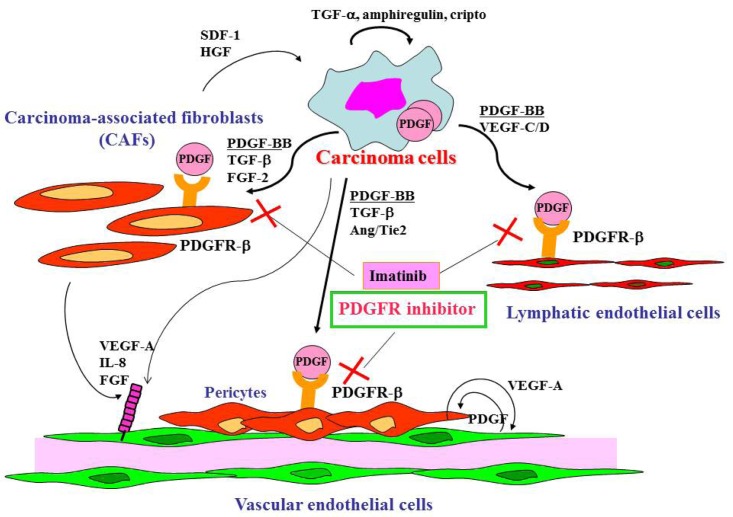
Gastric cancer cells express a variety of growth factors and cytokines, which act in an autocrine or paracrine manner. Interaction between tumor cells and stromal cells through the PDGF/PDGF receptor (PDGFR) system influences stromal reaction, lymphangiogenesis and maturation of tumor vessels. PDGFR tyrosine kinase inhibitor can modulate the tumor microenvironment and enhance the effect of chemotherapeutic agents.

Gastric cancer cells express a broad spectrum of growth factors, angiogenic factors, lymphangiogenic factors and cytokines (Figure 1). Members of the epidermal growth factor family, including transforming growth factor (TGF)-α, amphiregulin and cripto, act as autocrine growth factors for gastric cancer cells. Growth factors that regulate angiogenesis are vascular endothelial growth factor (VEGF)-A [41], interleukin-8 [42], fibroblast growth factor (FGF)-2 [43] and platelet-derived endothelial cell growth factor (PD-ECGF) [44]. These angiogenic factors are released not only by cancer cells but also by CAFs and inflammatory cells in tumor stroma. Lymphangiogenic factors such as VEGF-C [45] and -D [46] are also expressed by gastric carcinoma cells. TGF-β, PDGF, insulin-like growth factor (IGF)-II and FGF-2 are involved stroma reaction and commonly overexpressed in diffuse-type gastric cancers including scirrhous cancers. Development of scirrhous gastric cancer in particular may require synchronous overexpression of TGF-β, PDGF, IGF-II and FGF-2, all of which may function mainly as paracrine growth factors [6]. Although the mechanisms that regulate activation of fibroblasts and their accumulation in tumors are not fully understood, PDGF, TGF-β and FGF-2 are known to be involved in this process [16,17,47]. Recently, cancer exosomes were shown to trigger fibroblast differentiation into CAFs due to the presence of TGF-β at the exosome surface [48]. The crosstalk between tumor cells and stromal cells is bi-directional, with hepatocyte growth factor and stromal cell-derived factor-1 secreted by CAFs, stimulating the growth and progression of tumor cells themselves [49].

## 5. PDGF Associates with Stromal Reaction in Human Gastric Cancers

PDGF and PDGFR are expressed in many types of human neoplasm, including gastric cancer [50-52]. PDGF is a dimeric protein of the following molecular variants: PDGF-AA, PDGF-BB, PDGF-AB, PDGF-CC and PDGF-DD [53]. PDGFR signaling is reported to increase proliferation of tumor cells in an autocrine manner [54] and to stimulate angiogenesis [55], recruit pericytes [54,56] and control interstitial fluid pressure in stroma, influencing transvascular transport of chemotherapeutic agents in a paracrine manner [57].

Under culture conditions, gastric cancer cell lines express PDGF-B at various levels but not PDGFR-β [51]. When cells from these lines are implanted orthotopically into the gastric wall, cells with high PDGF expression produce tumors with abundant stroma. In contrast, cells with low PDGF-B expression form medullary tumors with little stromal reaction. In surgical specimens, we found expression of PDGF-B to be associated with stromal reaction [52]. We also found expression of PDGF-B and PDGFR-β to be significantly greater in diffuse-type gastric carcinomas than in intestinal-type gastric carcinomas [52].

In gastric cancer tissues, PDGF-B expression is found in tumor cells, but PDGFR-β expression is found predominantly in stromal cells. By double immunofluorescence, it has become apparent that PDGFR-β is expressed by CAFs, pericytes and lymphatic endothelial cells in stroma of gastric cancer tissues [51]. Pericytes play an important role in regulating vessel maturation and function by production of VEGF, which stabilizes endothelial cells. A variety of signaling factors mediate pericyte-endothelial cells interaction, including VEGF, PDGF-B and Ang/Tie2. We recently found correlation between the PDGF-B mRNA expression level in gastric carcinoma tissues and lymphatic metastasis, suggesting that PDGF-B acts as a lymphangiogenic factor [52].

## 6. Stroma-Directed Molecular Targeted Therapy

A novel category of anti-cancer drugs, “molecular-targeted drugs” has become available. The tumor microenvironment is increasingly being viewed as a potential therapeutic target, and strategies are being developed to disrupt tumor-stroma interactions. Although efficacy of anti-angiogenic therapy has been studied extensively, the concept of targeting CAFs to obtain a therapeutic advantage in cancer has not been explored in depth.

Angiogenesis is considered one of the most important molecular targets for anti-cancer therapy because it is essential for tumor growth and metastasis. Weidner *et al.* [58] first reported a direct correlation between the incidence of metastasis and the number and density of blood vessels in invasive breast cancers. Similar studies have confirmed this correlation in gastric cancers [41,59]. Induction of angiogenesis is mediated by various molecules released by both tumor and host cells [60]. Several growth factors that regulate angiogenesis have been identified. VEGF-A is one of the most potent angiogenic factors and is expressed in almost all human solid tumors, including gastrointestinal cancers [61-63]. In these cancers, expression of VEGF-A correlates with advanced-stage disease and poor prognosis. Therefore, inhibiting VEGF-A is a rational strategy for treating cancer. Bevacizumab is a humanized monoclonal antibody that targets VEGF-A. Significantly prolonged survival has been reported in patients with metastatic colorectal cancer treated with bevacizumab in combination with a cytotoxic agent(s) [64]. A randomized trial evaluating the efficacy of bevacizumab combination therapy in patients with gastric cancer (the AVAGAST study) was conducted internationally, Japan and Korea included. Adding bevacizumab to chemotherapy as first-line treatment for advanced gastric cancer did not significantly increase overall survival (the primary endpoint of the study) [65]. The reason AVAGAST did not achieve the primary objective is not clear, but it might have been due in part to the histologic heterogeneity of gastric cancer. The prognosis of gastric cancer depends on both histologic type and disease stage [66]. Intestinal-type gastric cancer tends to metastasize to the liver in a hematogenous manner. In contrast, diffuse-type gastric cancer is more invasive; dissemination is predominantly peritoneal. Factors responsible for liver metastasis and peritoneal dissemination have not yet been identified; however, we have found that the angiogenic phenotype differs between intestinal-type and diffuse-type gastric cancers. The intestinal-type is more dependent on angiogenesis. Intestinal-type, but not diffuse-type, tumors have been shown to express high levels of VEGF-A, and the VEGF-A expression level correlates significantly with vessel count [41,67]. In contrast, expression levels of PDGF, TGF-β and FGF-2 are higher in diffuse-type tumors, especially scirrhous-type tumors [43,50,68].

**Figure 2.**
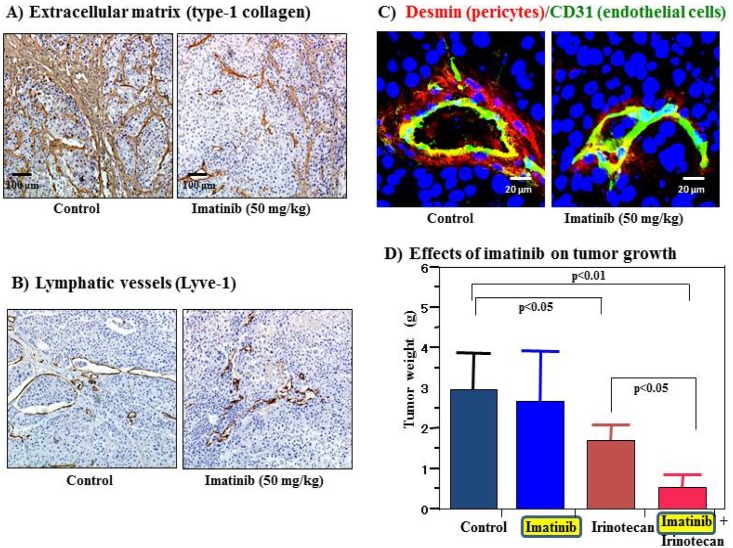
Effects of imatinib on extracellular matrix (ECM), lymphatic vessels, and pericytes. (**A**, **B**) Immunohistochemical detection of type-1 collagen (**A**) and lyve-1 in TMK-1 orthotopic tumors was carried out by the linked streptavidin-biotin method. The ECM and lymphatic vessel areas were reduced by treatment with imatinib; (**C**) Morphology and distribution of pericytes (as shown by red fluorescence) differs between tumors in control mice and tumors in mice treated with imatinib. Endothelial cells are shown by green fluorescence; (**D**) Antitumor effects of imatinib and irinotecan on the growth of TMK-1 orthotopic tumors in mice. Treatment with irinotecan alone significantly inhibited tumor growth in mice treated with irinotecan alone, and imatinib in combination with irinotecan enhanced the antitumor effects of irinotecan.

Cell surface receptors are feasible targets for cancer therapy, and several PDGFR inhibitors are used routinely in clinical practice. Imatinib, sorafenib and sunitinib are currently used for various malignancies, including gastrointestinal stromal tumor, chronic myeloid leukemia, hepatocellular carcinoma and renal cell carcinoma. These agents have been shown to target tumor cells and should naturally target PDGFR on CAFs and pericytes as well. We determined the effects of imatinib, PDGFR-β tyrosine kinase inhibitor, on stromal components in tumors grown up from human gastric carcinoma cells implanted into the stomachs of nude mice [51]. The stromal reaction was significantly reduced in mice treated with imatinib in comparison to that in control mice (Figure 2A). We also noted morphologic differences between pericytes in the control mice and pericytes in the imatinib-treated mice. Pericytes in the control mice were enlarged and overlapped each other, whereas pericytes in mice treated with imatinib were very thin (Figure 2C) [51]. However, stromal alteration by imatinib alone had no immediate anti-tumor effect in our experimental model. Therefore, irinotecan was administered with imatinib. We found that blockade of PDGFR-β signaling by oral administration of imatinib combined with intraperitoneal injection of irinotecan significantly inhibited not only the growth of tumors (Figure 2D) but also the incidences of lymph node and peritoneal metastasis. Blockade of PDGFR signaling decreased stromal reaction and the areas of vascular and lymphatic vessels (Figure 2B). Imatinib may inhibit pericyte coverage and disrupt interaction between pericytes and endothelial cells, sensitizing endothelial cells to chemotherapeutic agents. In addition, disruption of the reactive stroma by imatinib decreases interstitial fluid pressure and facilitates drug delivery, enhancing the efficacy of irinotecan in gastric cancer (Figure 1).

Recently, we found that tumor tropism of MSCs was inhibited by treatment with imatinib *in vivo* and *in vitro*. Oral administration of imatinib significantly inhibited the tumor growth- and metastasis-promoting effects of MSCs in our orthotopic colon cancer and liver metastasis models. Treatment with imatinib also decreased the number of MSCs in the tumor stroma and inhibited the cell proliferation- and angiogenesis-promoting effects of MSCs as well as the apoptosis-inhibiting effect of MSCs [69]. These findings point to a possibility that migration of MSCs and the tumor-promoting effects of MSCs can be controlled by molecularly targeted anti-tumor drugs aimed at bone marrow-derived cells.

In phase II clinical trials, imatinib monotherapy has been shown to be largely ineffective for malignant glioma, breast and prostate cancers [70-73]. Similarly, imatinib combined with cytotoxic chemotherapeutic agents has failed thus far [74,75]. The reasons are unclear, but the clinical trials to date have enrolled unselected patients. As noted above, gastric cancer cells express PDGF-B, but PDGFR-β expression is found predominantly in stromal cells. In our experimental animal models, treatment with imatinib alone or in combination with cytotoxic agents had no effect on the growth and metastasis of medullary tumors, for which the stromal reaction is minimal [51]. Stromal compartment-rich tumors, such as diffuse-type gastric carcinomas, may be tumors in which targeting the PDGF/PDGF-R signaling pathway for enhancement of the chemotherapeutic effect is most applicable.

## 7. Conclusions

The different cell types populating the tumor stroma, *i.e.*, CAFs, endothelial cells, pericytes and inflammatory cells, and the ECM help to create a microenvironment permissive of tumor growth, angiogenesis and invasion. The findings presented in this review indicate that stroma-directed molecular targeted therapy might be a valid complement to conventional treatments that target the cancer cells themselves. Because most solid tumors have reactive stroma, targeting stromal cells may have broad clinical implications as a therapeutic strategy. Further understanding of the cellular and molecular mechanisms that regulate cancer-stromal cell interaction and inhibition of stromal cell activation may facilitate development of an effective anti-tumor therapy.

## References

[1] Jemal A., Siegel R., Ward E., Hao Y., Xu J., Thun M.J. (2009). Cancer statistics, 2009. CA Cancer J. Clin..

[2] (1994). Schistosomes, liver flukes and Helicobacter pylori.

[3] Watanabe T., Tada M., Nagai H., Sasaki S., Nakao M. (1998). *Helicobacter pylori* infection induces gastric cancer in mongolian gerbils. Gastroenterology.

[4] Honda S., Fujioka T., Tokieda M., Satoh R., Nishizono A., Nasu M. (1998). Development of *Helicobacter pylori*-induced gastric carcinoma in Mongolian gerbils. Cancer Res..

[5] Smith M.G., Hold G.L., Tahara E., El-Omar E.M. (2006). Cellular and molecular aspects of gastric cancer. World J. Gastroenterol..

[6] Tahara E. (2004). Genetic pathways of two types of gastric cancer. IARC Sci. Publ..

[7] Mantovani A., Allavena P., Sica A., Balkwill F. (2008). Cancer-related inflammation. Nature.

[8] Whiteside T.L. (2008). The tumor microenvironment and its role in promoting tumor growth. Oncogene.

[9] Mueller M.M., Fusenig N.E. (2004). Friends or foes-bipolar effects of the tumour stroma in cancer. Nat. Rev. Cancer.

[10] Paunescu V., Bojin F.M., Tatu C.A., Gavriliuc O.I., Rosca A., Gruia A.T., Tanasie G., Bunu C., Crisnic D., Gherghiceanu M. (2011). Tumour-associated fibroblasts and mesenchymal stem cells: More similarities than differences. J. Cell. Mol. Med..

[11] Kalluri R., Zeisberg M. (2006). Fibroblasts in cancer. Nat. Rev. Cancer.

[12] Dvorak H.F. (1986). Tumors: Wounds that do not heal. Similarities between tumor stroma generation and wound healing. N. Engl. J. Med..

[13] Allinen M., Beroukhim R., Cai L., Brennan C., Lahti-Domenici J., Huang H., Porter D., Hu M., Chin L., Richardson A. (2004). Molecular characterization of the tumor microenvironment in breast cancer. Cancer Cell.

[14] Kuperwasser C., Chavarria T., Wu M., Magrane G., Gray J.W., Carey L., Richardson A., Weinberg R.A. (2004). Reconstruction of functionally normal and malignant human breast tissues in mice. Proc. Natl. Acad. Sci. USA.

[15] Bhowmick N.A., Chytil A., Plieth D., Gorska A.E., Dumont N., Shappell S., Washington M.K., Neilson E.G., Moses H.L. (2004). TGF-beta signaling in fibroblasts modulates the oncogenic potential of adjacent epithelia. Science.

[16] Orimo A., Gupta P.B., Sgroi D.C., Arenzana-Seisdedos F., Delaunay T., Naeem R., Carey V.J., Richardson A.L., Weinberg R.A. (2005). Stromal fibroblasts present in invasive human breast carcinomas promote tumor growth and angiogenesis through elevated SDF-1/CXCL12 secretion. Cell.

[17] de Wever O., Mareel M. (2003). Role of tissue stroma in cancer cell invasion. J. Pathol..

[18] Rodemann H.P., Muller G.A. (1991). Characterization of human renal fibroblasts in health and disease: II. *In vitro* growth, differentiation, and collagen synthesis of fibroblasts from kidneys with interstitial fibrosis. Am. J. Kidney Dis..

[19] Tomasek J.J., Gabbiani G., Hinz B., Chaponnier C., Brown R.A. (2002). Myofibroblasts and mechano-regulation of connective tissue remodelling. Nat. Rev. Mol. Cell Biol..

[20] Micke P., Ostman A. (2005). Exploring the tumour environment: Cancer-associated fibroblasts as targets in cancer therapy. Expert Opin. Ther. Targets.

[21] Tuxhorn J.A., Ayala G.E., Rowley D.R. (2001). Reactive stroma in prostate cancer progression. J. Urol..

[22] Moinfar F., Man Y.G., Arnould L., Bratthauer G.L., Ratschek M., Tavassoli F.A. (2000). Concurrent and independent genetic alterations in the stromal and epithelial cells of mammary carcinoma: Implications for tumorigenesis. Cancer Res..

[23] Lieubeau B., Garrigue L., Barbieux I., Meflah K., Gregoire M. (1994). The role of transforming growth factor beta 1 in the fibroblastic reaction associated with rat colorectal tumor development. Cancer Res..

[24] Henry L.R., Lee H.O., Lee J.S., Klein-Szanto A., Watts P., Ross E.A., Chen W.T., Cheng J.D. (2007). Clinical implications of fibroblast activation protein in patients with colon cancer. Clin. Cancer Res..

[25] Tsujino T., Seshimo I., Yamamoto H., Ngan C.Y., Ezumi K., Takemasa I., Ikeda M., Sekimoto M., Matsuura N., Monden M. (2007). Stromal myofibroblasts predict disease recurrence for colorectal cancer. Clin. Cancer Res..

[26] Hwang R.F., Moore T., Arumugam T., Ramachandran V., Amos K.D., Rivera A., Ji B., Evans D.B., Logsdon C.D. (2008). Cancer-associated stromal fibroblasts promote pancreatic tumor progression. Cancer Res..

[27] Anderberg C., Pietras K. (2009). On the origin of cancer-associated fibroblasts. Cell Cycle.

[28] Zeisberg E.M., Potenta S., Xie L., Zeisberg M., Kalluri R. (2007). Discovery of endothelial to mesenchymal transition as a source for carcinoma-associated fibroblasts. Cancer Res..

[29] Direkze N.C., Hodivala-Dilke K., Jeffery R., Hunt T., Poulsom R., Oukrif D., Alison M.R., Wright N.A. (2004). Bone marrow contribution to tumor-associated myofibroblasts and fibroblasts. Cancer Res..

[30] Pittenger M.F., Mackay A.M., Beck S.C., Jaiswal R.K., Douglas R., Mosca J.D., Moorman M.A., Simonetti D.W., Craig S., Marshak D.R. (1999). Multilineage potential of adult human mesenchymal stem cells. Science.

[31] Studeny M., Marini F.C., Dembinski J.L., Zompetta C., Cabreira-Hansen M., Bekele B.N., Champlin R.E., Andreeff M. (2004). Mesenchymal stem cells: Potential precursors for tumor stroma and targeted-delivery vehicles for anticancer agents. J. Natl. Cancer Inst..

[32] Psaila B., Lyden D. (2009). The metastatic niche: Adapting the foreign soil. Nat. Rev. Cancer.

[33] Shinagawa K., Kitadai Y., Tanaka M., Sumida T., Kodama M., Higashi Y., Tanaka S., Yasui W., Chayama K. (2010). Mesenchymal stem cells enhance growth and metastasis of colon cancer. Int. J. Cancer.

[34] Menon L.G., Picinich S., Koneru R., Gao H., Lin S.Y., Koneru M., Mayer-Kuckuk P., Glod J., Banerjee D. (2007). Differential gene expression associated with migration of mesenchymal stem cells to conditioned medium from tumor cells or bone marrow cells. Stem Cells.

[35] Dwyer R.M., Potter-Beirne S.M., Harrington K.A., Lowery A.J., Hennessy E., Murphy J.M., Barry F.P., O'Brien T., Kerin M.J. (2007). Monocyte chemotactic protein-1 secreted by primary breast tumors stimulates migration of mesenchymal stem cells. Clin. Cancer Res..

[36] Beckermann B.M., Kallifatidis G., Groth A., Frommhold D., Apel A., Mattern J., Salnikov A.V., Moldenhauer G., Wagner W., Diehlmann A. (2008). VEGF expression by mesenchymal stem cells contributes to angiogenesis in pancreatic carcinoma. Br. J. Cancer.

[37] Mishra P.J., Mishra P.J., Humeniuk R., Medina D.J., Alexe G., Mesirov J.P., Ganesan S., Glod J.W., Banerjee D. (2008). Carcinoma-associated fibroblast-like differentiation of human mesenchymal stem cells. Cancer Res..

[38] Guo X., Oshima H., Kitmura T., Taketo M.M., Oshima M. (2008). Stromal fibroblasts activated by tumor cells promote angiogenesis in mouse gastric cancer. J. Biol. Chem..

[39] Worthley D.L., Ruszkiewicz A., Davies R., Moore S., Nivison-Smith I., Bik To L., Browett P., Western R., Durrant S., So J. (2009). Human gastrointestinal neoplasia-associated myofibroblasts can develop from bone marrow-derived cells following allogeneic stem cell transplantation. Stem Cells.

[40] Quante M., Tu S.P., Tomita H., Gonda T., Wang S.S., Takashi S., Baik G.H., Shibata W., Diprete B., Betz K.S. (2011). Bone marrow-derived myofibroblasts contribute to the mesenchymal stem cell niche and promote tumor growth. Cancer Cell.

[41] Takahashi Y., Cleary K.R., Mai M., Kitadai Y., Bucana C.D., Ellis L.M. (1996). Significance of vessel count and vascular endothelial growth factor and its receptor (KDR) in intestinal-type gastric cancer. Clin. Cancer Res..

[42] Kitadai Y., Haruma K., Sumii K., Yamamoto S., Ue T., Yokozaki H., Yasui W., Ohmoto Y., Kajiyama G., Fidler I.J. (1998). Expression of interleukin-8 correlates with vascularity in human gastric carcinomas. Am. J. Pathol..

[43] Tanimoto H., Yoshida K., Yokozaki H., Yasui W., Nakayama H., Ito H., Ohama K., Tahara E. (1991). Expression of basic fibroblast growth factor in human gastric carcinomas. Virchows Arch. B Cell Pathol. Incl. Mol. Pathol..

[44] Takahashi Y., Bucana C.D., Akagi Y., Liu W., Cleary K.R., Mai M., Ellis L.M. (1998). Significance of platelet-derived endothelial cell growth factor in the angiogenesis of human gastric cancer. Clin. Cancer Res..

[45] Amioka T., Kitadai Y., Tanaka S., Haruma K., Yoshihara M., Yasui W., Chayama K. (2002). Vascular endothelial growth factor-C expression predicts lymph node metastasis of human gastric carcinomas invading the submucosa. Eur. J. Cancer.

[46] Onogawa S., Kitadai Y., Tanaka S., Kuwai T., Kimura S., Chayama K. (2004). Expression of VEGF-C and VEGF-D at the invasive edge correlates with lymph node metastasis and prognosis of patients with colorectal carcinoma. Cancer Sci..

[47] Pietras K., Sjoblom T., Rubin K., Heldin C.H., Ostman A. (2003). PDGF receptors as cancer drug targets. Cancer Cell.

[48] Webber J., Steadman R., Mason M.D., Tabi Z., Clayton A. (2010). Cancer exosomes trigger fibroblast to myofibroblast differentiation. Cancer Res..

[49] van Zijl F., Mair M., Csiszar A., Schneller D., Zulehner G., Huber H., Eferl R., Beug H., Dolznig H., Mikulits W. (2009). Hepatic tumor-stroma crosstalk guides epithelial to mesenchymal transition at the tumor edge. Oncogene.

[50] Tsuda T., Yoshida K., Tsujino T., Nakayama H., Kajiyama G., Tahara E. (1989). Coexpression of platelet-derived growth factor (PDGF) A-chain and PDGF receptor genes in human gastric carcinomas. Cancer Sci..

[51] Sumida T., Kitadai Y., Shinagawa K., Tanaka M., Kodama M., Ohnishi M., Ohara E., Tanaka S., Yasui W., Chayama K. (2011). Anti-stromal therapy with imatinib inhibits growth and metastasis of gastric carcinoma in an orthotopic nude mouse model. Int. J. Cancer.

[52] Kodama M., Kitadai Y., Sumida T., Ohnishi M., Ohara E., Tanaka M., Shinagawa K., Tanaka S., Yasui W., Chayama K. (2010). Expression of platelet-derived growth factor (PDGF)-B and PDGF-receptor beta is associated with lymphatic metastasis in human gastric carcinoma. Cancer Sci..

[53] Bergsten E., Uutela M., Li X., Pietras K., Ostman A., Heldin C.H., Alitalo K., Eriksson U. (2001). PDGF-D is a specific, protease-activated ligand for the PDGF beta-receptor. Nat. Cell Biol..

[54] Ostman A. (2004). PDGF receptors-mediators of autocrine tumor growth and regulators of tumor vasculature and stroma. Cytokine Growth Factor Rev..

[55] Risau W., Drexler H., Mironov V., Smits A., Siegbahn A., Funa K., Heldin C.H. (1992). Platelet-derived growth factor is angiogenic *in vivo.*. Growth Factors.

[56] Bergers G., Song S., Meyer-Morse N., Bergsland E., Hanahan D. (2003). Benefits of targeting both pericytes and endothelial cells in the tumor vasculature with kinase inhibitors. J. Clin. Invest..

[57] Pietras K. (2004). Increasing tumor uptake of anticancer drugs with imatinib. Semin. Oncol..

[58] Weidner N., Semple J.P., Welch W.R., Folkman J. (1991). Tumor angiogenesis and metastasis—Correlation in invasive breast carcinoma. N. Engl. J. Med..

[59] Tanigawa N., Amaya H., Matsumura M., Shimomatsuya T., Horiuchi T., Muraoka R., Iki M. (1996). Extent of tumor vascularization correlates with prognosis and hematogenous metastasis in gastric carcinomas. Cancer Res..

[60] Folkman J. (1986). How is blood vessel growth regulated in normal and neoplastic tissue? G.H.A. Clowes memorial Award lecture. Cancer Res..

[61] Kitadai Y., Haruma K., Tokutomi T., Tanaka S., Sumii K., Carvalho M., Kuwabara M., Yoshida K., Hirai T., Kajiyama G. (1998). Significance of vessel count and vascular endothelial growth factor in human esophageal carcinomas. Clin. Cancer Res..

[62] Takahashi Y., Kitadai Y., Bucana C.D., Cleary K.R., Ellis L.M. (1995). Expression of vascular endothelial growth factor and its receptor, KDR, correlates with vascularity, metastasis, and proliferation of human colon cancer. Cancer Res..

[63] Maeda K., Chung Y.S., Ogawa Y., Takatsuka S., Kang S.M., Ogawa M., Sawada T., Sowa M. (1996). Prognostic value of vascular endothelial growth factor expression in gastric carcinoma. Cancer.

[64] Hurwitz H., Fehrenbacher L., Novotny W., Cartwright T., Hainsworth J., Heim W., Berlin J., Baron A., Griffing S., Holmgren E. (2004). Bevacizumab plus irinotecan, fluorouracil, and leucovorin for metastatic colorectal cancer. N. Engl. J. Med..

[65] Ohtsu A., Shah M.A., van Cutsem E., Rha S.Y., Sawaki A., Park S.R., Lim H.Y., Yamada Y., Wu J., Langer B. (2011). Bevacizumab in combination with chemotherapy as first-line therapy in advanced gastric cancer: A randomized, double-blind, placebo-controlled phase III study. J. Clin. Oncol..

[66] Duarte I., Llanos O. (1981). Patterns of metastases in intestinal and diffuse types of carcinoma of the stomach. Hum. Pathol..

[67] Yamamoto S., Yasui W., Kitadai Y., Yokozaki H., Haruma K., Kajiyama G., Tahara E. (1998). Expression of vascular endothelial growth factor in human gastric carcinomas. Pathol. Int..

[68] Yoshida K., Yokozaki H., Niimoto M., Ito H., Ito M., Tahara E. (1989). Expression of TGF-beta and procollagen type I and type III in human gastric carcinomas. Int. J. Cancer.

[69] Shinagawa K., Kitadai Y. (2011).

[70] Morris P.G., Abrey L.E. (2010). Novel targeted agents for platelet-derived growth factor receptor and c-KIT in malignant gliomas. Target. Oncol..

[71] Rao K., Goodin S., Levitt M.J., Dave N., Shih W.J., Lin Y., Capanna T., Doyle-Lindrud S., Juvidian P., DiPaola R.S. (2005). A phase II trial of imatinib mesylate in patients with prostate specific antigen progression after local therapy for prostate cancer. Prostate.

[72] Bajaj G.K., Zhang Z., Garrett-Mayer E., Drew R., Sinibaldi V., Pili R., Denmeade S.R., Carducci M.A., Eisenberger M.A., DeWeese T.L. (2007). Phase II study of imatinib mesylate in patients with prostate cancer with evidence of biochemical relapse after definitive radical retropubic prostatectomy or radiotherapy. Urology.

[73] Modi S., Seidman A.D., Dickler M., Moasser M., D'Andrea G., Moynahan M.E., Menell J., Panageas K.S., Tan L.K., Norton L. (2005). A phase II trial of imatinib mesylate monotherapy in patients with metastatic breast cancer. Breast Cancer Res. Treat..

[74] Yardley D.A., Burris H.A., Markus T., Spigel D.R., Greco F.A., Mainwaring M., Waterhouse D.M., Webb C.D., Hainsworth J.D. (2009). Phase II trial of docetaxal plus imatinib mesylate in the treatment of patients with metastatic breast cancer. Clin. Breast Cancer.

[75] Chew H.K., Barlow W.E., Albain K., Lew D., Gown A., Hayes D.F., Gralow J., Hortobagyi G.N., Livingston R. (2008). A phase II study of imatinib mesylate and capecitabine in metastatic breast cancer: Southwest Oncology Group Study 0338. Clin. Breast Cancer.

